# Cost-Effectiveness Analysis of Baseline Testing for Resistance-Associated Polymorphisms to Optimize Treatment Outcome in Genotype 1 Noncirrhotic Treatment-Naïve Patients With Chronic Hepatitis C Virus

**DOI:** 10.1016/j.jval.2019.08.012

**Published:** 2020-02

**Authors:** Christopher G. Fawsitt, Peter Vickerman, Graham S. Cooke, STOP HCV Consortium, Eleanor Barnes, Jonathan Ball, Diana Brainard, Gary Burgess, Graham S. Cooke, John Dillon, Graham Foster, Charles Gore, Neil Guha, Rachel Halford, Kevin Whitby, Chris Holmes, Anita Howe, Emma Hudson, Sharon Hutchinson, William Irving, Salim Khakoo, Paul Klenerman, Natasha Martin, Benedetta Massetto, Tamyo Mbisa, John McHutchison, Jane McKeating, John McLauchlan, Alec Miners, Andrea Murray, Peter Shaw, Peter Simmonds, Chris Spencer, Emma Thomson, Peter Vickerman, Nicole Zitzmann, Nicky J. Welton

**Affiliations:** 1 Department of Population Health Sciences, Bristol Medical School, University of Bristol, Bristol, England, UK; 2 Department of Medicine, Imperial College London, London, England, UK

**Keywords:** baseline testing, cost-effectiveness, hepatitis C virus, resistance-associated polymorphisms

## Abstract

**Objectives:**

Direct-acting antivirals containing nonstructural protein 5A (NS5A) inhibitors administered over 8 to 12 weeks are effective in ∼95% of patients with hepatitis C virus. Nevertheless, patients resistant to NS5A inhibitors have lower cure rates over 8 weeks (<85%); for these patients, 12 weeks of treatment produces cure rates greater than 95%. We evaluated the lifetime cost-effectiveness of testing for NS5A resistance at baseline and optimizing treatment duration accordingly in genotype 1 noncirrhotic treatment-naïve patients from the perspective of the UK National Health Service.

**Methods:**

A decision-analytic model compared (1) standard 12-week treatment (no testing), (2) shortened 8-week treatment (no testing), and (3) baseline testing with 12-/8-week treatment for those with/without NS5A polymorphisms. Patients who failed first-line therapy were retreated for 12 weeks. Model inputs were derived from published studies. Costs, quality-adjusted life-years, and the probability of cost-effectiveness were calculated.

**Results:**

Baseline testing had an incremental net monetary benefit (INMB) of £11 838 versus standard 12 weeks of therapy (no testing) and low probability (31%) of being the most cost-effective, assuming £30 000 willingness to pay. Shortened 8 weeks of treatment (no testing) had an INMB of £12 294 and the highest probability (69%) of being most cost-effective. Scenario analyses showed baseline testing generally had the highest INMB and probability of being most cost-effective if first- and second-line drug prices were low (<£20k).

**Conclusions:**

Optimizing treatment duration based on NS5A polymorphisms for genotype 1 noncirrhotic treatment-naive patients in the United Kingdom is not cost-effective if the drug costs are high; the strategy is generally most cost-effective when drug prices are low (<£20k).

## Highlights

• Although direct-acting antivirals containing nonstructural protein 5A (NS5A) inhibitors administered over 8 to 12 weeks are effective in ∼95% of patients with hepatitis C virus (HCV), the licensed duration of therapy may be unnecessary or less effective for selected patients. Patients with resistance to NS5A inhibitors have lower cure rates over 8 weeks and would benefit more from standard 12-week treatment. Conversely, nonresistant patients generally do not require 12-week treatment; 8-week treatment produces comparable cure rates. Testing for resistance to NS5A inhibitors at baseline and optimizing treatment duration accordingly is one method that could be used to improve patient outcomes. Nevertheless, the potential benefits of resistance testing must be considered in the context of the added cost of the resistance test.
• This is the first study to consider the cost-effectiveness of baseline testing in genotype 1 noncirrhotic treatment-naïve patients. Furthermore, it is the first to consider it in the context of a shortened treatment duration of 8 weeks, which has been shown to be cost-effective versus standard 12-week treatment for many commonly used direct-acting antivirals.
• Baseline testing has a low probability of being the most cost-effective treatment strategy if first- and second-line drug prices per 12-week course are high (>£20k). Nevertheless, if drug costs are low (<£20k), baseline testing generally has the highest probability of being the most cost-effective strategy; it also has the highest probability of being most cost-effective if the cost of first-line treatment is low (<£20k) and second-line treatment is high (>£22k).

## Introduction

The burden and prevalence of hepatitis C virus (HCV) worldwide remains high, with more than 70 million people, or 1% of the world’s population, currently living with the chronic infection.^1^ The World Health Organization recently committed to reducing the number of new HCV cases and deaths worldwide by 2030.^2^ Efforts to reduce the burden of HCV have been invigorated by the advent of direct-acting antivirals (DAAs), which produce high cure rates (∼95%) over relatively short courses of treatment (8-12 weeks) and offer good side-effect profiles.^3^ Nevertheless, there is emerging evidence that, for selected patients, treatment over the licensed duration of therapy can be unnecessary or less effective. DAA regimens containing nonstructural protein 5A (NS5A) inhibitor can be less effective in patients with NS5A polymorphisms or resistance-associated substitutions (RASs). One widely used combination therapy, ledipasvir/sofosbuvir (LDV/SOF), for example, produces high and relatively comparable cure rates or sustained virological response (SVR, effective cure) over a standard 12-week treatment duration (96.3%)^4^ as a shortened 8-week treatment duration (94.6%) in genotype 1 (GT1) noncirrhotic treatment-naïve (TN) patients.^5^ Nevertheless, in NS5A inhibitor-resistant patients, a significantly lower SVR has been observed over an 8-week treatment duration (82.8%) than over a 12-week treatment duration (95.7%).^5^ Outcomes for this group could be considerably improved if the patient’s resistance profile was determined at baseline using single-gene sequencing, or resistance testing, and treatment duration optimized accordingly.

Despite the clinical benefits resistance testing can provide, it is not widely used. In some circumstances, resistance testing is recommended routinely when optimizing treatment for an individual patient. For one combination therapy, elbasvir/grazoprevir (ELB/GZR), resistance testing is recommended to guide the duration of therapy.^6^ In patients with NS5A polymorphisms, a 16-week treatment duration is recommended, whereas a standard 12-week treatment duration is recommended for patients without the RASs. The cost-effectiveness of resistance testing has also been documented in the literature.^7^^,^^8^ Westerhout et al^8^ considered the cost-effectiveness of testing for NS5A polymorphisms at baseline in GT1 treatment-experienced patients with severe or compensated cirrhosis in Italy. Patients were treated for 12 weeks if they had severe cirrhosis and NS5A polymorphisms at baseline and 24 weeks if they had compensated cirrhosis and NS5A polymorphisms at baseline. The authors found baseline testing was cost-effective versus no testing (with nonstratified treatment durations of 12 or 24 weeks for patients with severe or compensated cirrhosis, respectively).^8^ In the United States, Elbasha and colleagues^7^ considered the cost-effectiveness of baseline testing in GT1a TN and treatment-experienced patients. Nevertheless, the authors treated patients for 12 weeks if no NS5A RASs were present at baseline and 16 weeks otherwise. The authors similarly found the results favored baseline testing versus no testing in noncirrhotic TN patients.^7^ No study has yet considered the cost-effectiveness of baseline testing in the context of shorter treatment durations, which have been shown to be highly clinically effective^5^^,^^9^ and cost-effective^10^ in GT1 noncirrhotic TN patients.

Adjusting the treatment duration based on the presence of NS5A polymorphisms carries the potential to increase the rate of successful outcomes in patients through increased cure rates, thereby limiting the incidence of liver-related morbidity and mortality and associated healthcare costs. Nevertheless, baseline testing introduces additional costs that must be considered in the context of its potential benefit. In this article, we investigate the lifetime cost-effectiveness of testing for resistance to NS5A inhibitor-containing regimens at baseline in GT1 noncirrhotic TN patients in the United Kingdom, with treatment duration optimized to 12 weeks in NS5A-resistant patients and 8 weeks otherwise. We compared baseline testing against a standard 12-week treatment duration for all patients, which is the generally recommended treatment duration. An additional strategy of a shortened 8-week treatment duration for all patients was also considered because this strategy is sometimes recommended, particularly for newer regimens,^11^ and it may offer cost advantages beyond baseline testing that need to be considered.

## Methods

We adapted a previously validated decision tree and Markov model^10^ to assess the cost-effectiveness of baseline testing for NS5A polymorphisms from the perspective of the UK National Health Service (NHS). We assumed monthly cycles in the decision tree to simulate treatment outcomes in the first year and annual cycles in the Markov model to simulate the natural disease history of HCV. We adopted a lifetime time horizon, projecting outcomes over 60 years, and discounted future costs and benefits at 3.5% per annum, in line with UK guidance.^12^

### Patient Population

We modeled outcomes for HCV GT1 (subtypes 1a and 1b combined) noncirrhotic TN patients in the United Kingdom. We considered outcomes for patients with mild (F0-F1) and moderate (F2-F3) liver fibrosis, as informed by UK data^13^ (Table 1). Patients were aged 40 years, and 70% were male at model entry.^13^

**Table 1:** Summary of treatment, epidemiological, cost, and quality-of-life inputs for probabilistic sensitivity analyses.

Variable	Base case	Distribution	Alpha∗	Beta∗	Source
Patient characteristics					
Initial distribution of liver fibrosis					
Mild (F0-F1)	51.1%	—	—	—	^13^
Moderate (F2-F3)	48.9%	—	—	—	^13^
Age	40	—	—	—	^13^
Male	70%	—	—	—	^13^
Efficacy (SVR12)					
First-line treatment: LDV/SOF					
NoTest12wks	0.963	Beta	208	8	^4^
NoTest8wks	0.946	Beta	209	12	^5^
Test12/8wks					
NS5A (12 weeks)	0.957	Beta	45	2	^5^
No NS5A (8 weeks)	0.964	Beta	185	7	^5^
Retreatment – SOF/VEL/VOX					
NoTest12wks//NoTest8wks	0.973	Beta	142	4	^14^
NS5A (Test12/8wks)	0.968	Beta	120	4	^14^
No NS5A (Test12/8wks)	0.977	Beta	42	1	^14^
Resistance prevalence					
NS5A	0.115	Beta	102	785	^5^
Annual transition probabilities					
Fibrosis progression					
Mild-to-moderate	0.025	Beta	38	1484	^15^^,^^16^
Moderate-to-CC	0.037	Beta	27	699	^15^^,^^16^
Nonfibrosis progression					
CC-to-DCC	0.039	Beta	15	359	^17^
CC-to-HCC	0.014	Beta	2	135	^18^
DCC-to-HCC	0.014	Beta	2	135	^18^
HCC-to-liver transplant	0.020	Beta	98	4801	^13^
DCC-to-liver transplant	0.020	Beta	98	4801	^15^
Liver-related mortality					
DCC-to-liver death	0.130	Beta	147	983	^17^
HCC-to-liver death (first year)	0.430	Beta	117	155	^17^
HCC-to-liver death (subsequent year)	0.430	Beta	117	155	^17^
Liver transplant-to-liver death (first year)	0.150	Beta	85	481	^15^
Liver transplant-to-liver death (subsequent year)	0.057	Beta	85	1407	^19^
Reinfection	0.010	Beta	4	391	^20^
Costs					
Resistance test costs					
Single-gene sequencing	£100.00	Fixed	—	—	Assumption
Treatment-related costs					
LDV/SOF (monthly)	£13 225.20	Fixed	—	—	^4^
SOF/VEL/VOX (monthly)	£14 942.33	Fixed	—	—	^21^
Monitoring costs (monthly)	£162.34	Fixed	—	—	^4^
Health state costs					
SVR mild (F0-F1)	£60.36	Gamma	34	2	^21^
SVR moderate (F2-F3)	£60.36	Gamma	34	2	^21^
Mild (F0-F1)	£166.50	Gamma	13	13	^13^
Moderate (F2-F3)	£612.50	Gamma	35	17	^21^
CC (F4)	£951.13	Gamma	17	54	^21^
DCC	£12 833.96	Gamma	15	849	^13^
HCC (first year)	£11 436.41	Gamma	13	894	^13^
HCC (subsequent year)	£11 436.41	Gamma	13	894	^13^
Liver transplant (first year)	£51 769.79	Gamma	15	3473	^13^
Liver transplant (subsequent year)	£1949.08	Gamma	14	136	^13^
Adverse event costs					
Anemia	£501.58	Gamma	10	48	^20^
Rash	£166.50	Gamma	16	10	^20^
Depression	£414.17	Gamma	16	26	^20^
Neutropenia	£980.26	Gamma	10	98	^20^
Thrombocytopenia	£875.16	Gamma	14	62	^20^
Utilities					
Treatment-related utilities (penalties)					
Mild (F0-F1) (monthly)	–0.002	Beta	72	39 466	^23^
Moderate (F2-F3) (monthly)	–0.002	Beta	72	39 466	^23^
Health state utilities					
SVR mild (F0-F1)	0.820	Fixed	—	—	^16^
SVR moderate (F2-F3)	0.710	Fixed	—	—	^16^
Mild (F0-F1)	0.770	Beta	141	42	^16^
Moderate (F2-F3)	0.660	Log-normal	—	—	^16^
CC (F4)	0.550	Log-normal	—	—	^16^
DCC	0.450	Beta	55	67	^16^
HCC (first year)	0.450	Beta	55	67	^16^
HCC (subsequent year)	0.450	Beta	55	67	^16^
Liver transplant (first year)	0.450	Beta	55	67	^13^
Liver transplant (subsequent year)	0.670	Beta	32	16	^16^

CC indicates compensated cirrhosis; DCC, decompensated cirrhosis; HCC, hepatocellular carcinoma; LDV/SOF, ledipasvir/sofosbuvir; SOF/VEL/VOX, sofosbuvir/velpatasvir/voxilaprevir; SVR12, sustained virological response at 12 weeks. *NoTest12wks*, standard 12-week treatment duration (with no testing); *NoTest8wks*, shortened 8-week treatment duration (with no testing); *Test12/8wks*, baseline testing with 12-week treatment duration if NS5A resistant, 8 weeks otherwise.

∗ Parameters of a beta distribution describing uncertainty in probability parameters.

### Treatment Strategies

We compared the following strategies:

• ***NoTest12wks***: standard 12-week treatment duration (with no testing)
• ***NoTest8wks***: shortened 8-week treatment duration (with no testing)
• ***Test12/8wks***: baseline testing with 12-week treatment duration if NS5A resistant, 8 weeks otherwise

For the purposes of this analysis, we assumed *NoTest12wks* as the reference strategy because this is the standard recommended treatment duration in the United Kingdom. Under each strategy, we assumed that patients who failed first-line treatment were retreated for 12 weeks, as per recent UK guidelines.^24^^,^^25^

We assumed LDV/SOF as first-line therapy because it may be recommended for use over 8 to 12 weeks in GT1 noncirrhotic TN patients, so there is considerable evidence available on the effectiveness of the regimen in the studied population over the different treatment durations. LDV/SOF is an NS5A inhibitor-containing regimen that is administered daily using a fixed-dose tablet; each tablet contains 90 mg LDV (NS5A inhibitor) and 400 mg SOF (polymerase inhibitor).^4^ We assumed sofosbuvir/velpatasvir/voxilaprevir (SOF/VEL/VOX) as second-line therapy (ie, retreatment regimen) because it is the currently recommended treatment regimen in patients who previously failed first-line therapy in the United Kingdom.^24^ SOF/VEL/VOX is also an NS5A inhibitor-containing regimen that is administered once daily using a fixed-dose tablet; each tablet contains 400 mg SOF, 100 mg VEL (NS5A inhibitor), and 100 mg VOX (protease inhibitor).^21^

### Model Structure

We used a decision tree and Markov model (see Appendix 1 in the Supplemental Materials found at https://doi.org/10.1016/j.jval.2019.08.012) to assess treatment and lifetime outcomes, respectively. Patients were treated for either 12 or 8 weeks, depending on the strategy, and they were assessed 12 weeks after end of treatment for an effective cure (SVR12). SVR12 was defined as having HCV ribonucleic acid (RNA) less than 25 IU/mL. A 12-week salvage regimen was administered at 24 weeks during the decision tree if patients failed first-line therapy.

A Markov model was used to reflect long-term outcomes beyond the decision tree. All patients entered the model based on their response to treatment (SVR or fail) and initial liver fibrosis (mild or moderate). HCV-cleared patients could become reinfected at any point during the model, whereas HCV-infected patients could progress to more advanced stages of liver disease, including compensated cirrhosis, decompensated cirrhosis, and hepatocellular carcinoma. Patients in these advanced health states were at risk of requiring a liver transplant. The model captured the varying risk of liver-related mortality in these advanced health states, with additional health states included in the hepatocellular carcinoma and liver transplant health states to reflect the initial and subsequent risk of mortality. The model also captured the risk of all-cause mortality.

### Model Assumptions

During treatment, we assumed patients could not progress to more advanced stages of liver disease. Patients who failed first-line treatment were retreated at 24 weeks during the decision tree. There are no guidelines on when a salvage treatment should be administered, and it is unclear whether the timing of retreatment affects patients’ chance of viral eradication. In our model, we assumed the timing did not affect retreatment success. Although HCV-cleared patients could become reinfected, to be conservative we assumed these patients were not treated again and progressed through the model. We applied drug costs on a per-tablet basis (estimated monthly), rather than a per-treatment success/failure basis.

### Parameter Inputs

Model inputs are presented in Table 1 and described below.

#### Treatment-Related Inputs

The primary source of evidence on NS5A prevalence and first-line treatment efficacy was derived from Sarrazin et al,^5^ who recently synthesized evidence from phase II and III clinical trials in Europe and the United States. The authors reported outcomes for 2144 patients who had been treated over 12 or 8 weeks using LDV/SOF. At baseline, 11.5% of GT1 noncirrhotic TN patients had at least 1 RAS that conferred more than 100-fold resistance to NS5A inhibitor using a 1% cutoff value for deep sequencing (Table 1). The RASs included Q30H, Q30G, Q30R, L31I, L31M, L31V, P32L, M28A, M28G, Q30E, Q30K, H58D, Y93C, Y93H, Y93N, and Y93S in GT1a and P58D, A92K, and Y93Hin GT1b. Overall, 94.6% of patients (including both those with and without NS5A polymorphisms) achieved SVR12 over 8 weeks. Patients with NS5A resistance at baseline had similar SVR12 over 12 weeks at 95.7% as patients without the RAS treated for 8 weeks at 96.4%. We used these prevalence and efficacy data and assumed beta distributions for *NoTest8wks* and *Test12/8wks*, with uncertainty around these estimates given in Table 1. In the United Kingdom, SVR12 in patients treated for a standard 12-week treatment duration using the same regimen is 96.3%.^4^ We used this efficacy source and assumed a beta distribution for *NoTest12wks*, with uncertainty in this parameter also described in Table 1.

Bourliere and colleagues^14^ provided evidence on the efficacy of retreatment using SOF/VEL/VOX over 12 weeks from two phase III clinical trials (POLARIS-1 and POLARIS-4). Overall, 97.3% of patients (142 of 146) achieved SVR12; this informed beta distributions on SVR12 in patients who failed either the *NoTest12wks* or *NoTest8wks* strategy. In patients with and without NS5A polymorphisms at baseline, 96.8% (120 of 124) and 97.7% (42 of 43) achieved SVR12, respectively; we used these data to inform beta distributions on SVR12 for *Test12/8wks* (Table 1).

We modeled the probability that treatment-related adverse events occur to reflect the potential impact of clinical events over different treatment durations. We used data from earlier work,^10^ which in turn was derived from Johnson and colleagues.^20^ We estimated the probability of adverse events over 12 and 8 weeks, respectively.^10^ Although these events were observed for an alternative DAA, we assumed the probabilities were comparable because side effect profiles are generally good across DAAs and occur uniformly.^26^ We applied the same probabilities for retreatment as first-line treatment. The events included anemia, rash, depression, grade 3 or 4 neutropenia, and grades 3 or 4 thrombocytopenia.

### Epidemiological Inputs

The natural disease history of HCV was modeled using the Markov model and annual transition probabilities, which are presented in Table 1. The parameter estimates were taken from published studies on the probability of reinfection (1% per annum),^20^ fibrosis^15^^,^^16^ and nonfibrosis^17^^,^^18^ progression, liver-related mortality,^15^^,^^17^^,^^19^ and all-cause mortality, stratified by age and sex.^27^

#### Costs

The model considered treatment-related and health state costs from the perspective of the UK NHS (Table 1). Treatment-related costs included resistance test costs, drug costs, monitoring costs,^4^ and adverse event costs.^20^ We assumed that the cost of a resistance test (single-gene sequencing) was £100 in the base case analysis. The cost of first-line treatment and retreatment was derived from the UK technology appraisals for LDV/SOF^4^ and SOF/VEL/VOX,^21^ respectively. We assumed resistance test costs, drug costs, and monitoring costs were fixed in the model; these were not expected to vary in the UK. Health state costs were derived from published studies in the United Kingdom.^13^^,^^22^ We valued costs at 2017-2018 prices, expressed in Sterling (£), and inflated any outdated prices to current prices.^28^ A gamma distribution was assumed for all nonfixed cost inputs.

#### Utilities and quality-adjusted life-years

We used quality-adjusted life-years (QALYs) as our measure of health benefit and calculated these using published utility estimates.^16^^,^^23^ Treatment-related utilities, which reflect the deterioration in quality of life owing to adverse events, were derived from Chahal and colleagues^23^ for first-line treatment (Table 1). We assumed the same utility penalties for retreatment as there were no published estimates available for the new salvage regimen (SOF/VEL/VOX) at the time of writing. Health state utilities were derived from Wright and colleagues.^16^ We assumed HCV-cleared patients had a higher utility than HCV-infected patients by a score of 0.05, consistent with previous analyses.^13^^,^^20^ We set this utility as fixed in the model and assumed beta distributions for all other utility parameters (Table 1).

### Cost-Effectiveness Analyses

#### Base-case analysis

We compared the lifetime cost-effectiveness of *Test12/8wks* and *NoTest8wks* against *NoTest12wks* for GT1 noncirrhotic TN patients in the United Kingdom. We calculated the expected lifetime costs and QALYs per 1000 patients using probabilistic sensitivity analysis. We performed 10 000 Monte Carlo simulations, with point estimates randomly sampled from predefined probability distributions, using Microsoft Excel software (Redmond, WA). We report the expected net monetary benefit (NMB), and relative to *NoTest12wks*, we calculated the expected incremental net monetary benefit (INMB) and 95% credible intervals (CrIs) using willingness-to-pay thresholds of £20 000 and £30 000. The optimal strategy at a given willingness-to-pay threshold is the strategy with the highest expected INMB (or equivalently expected NMB). We explored uncertainty in the optimal strategy by reporting the probability that each strategy was the most cost-effective strategy in a cost-effectiveness acceptability curve (CEAC), and the probability that the optimal strategy was the most cost-effective in a cost-effectiveness acceptability frontier (CEAF), both plotted across a range of different willingness-to-pay thresholds. We also reported the number of liver-related events (ie, decompensated cirrhosis, hepatocellular carcinoma, liver transplant) in each strategy and calculated the number of events avoided in the *NoTest8wks* and *Test12/8wks* strategies relative to *NoTest12wks*.

#### Sensitivity and scenario analyses

The prices paid for DAA regimens are not published. To take account of lower prices negotiated as part of large-volume deals, we investigated a price reduction to the DAA regimens of 80%, which lowered the cost of first-line treatment (LDV/SOF) and retreatment (SOF/VEL/VOX) to £7935 and £8965 per patient over 12 weeks, respectively. We assumed this overall reduction to allow for differences in the cost of first- and second-line therapy, as SOF/VEL/VOX was expected to cost more than LDV/SOF.

Assuming the same 80% reduction in drug costs, we conducted a range of other sensitivity and scenario analyses. We undertook a 1-way deterministic sensitivity analysis of *NoTest8wks* and *Test12/8wks* relative to *NoTest12wks*, respectively, to investigate how sensitive the results were to fluctuations in parameter values. Where we had limited data on parameters, such as the cost of first- and second-line treatment, we assessed uncertainty using ±20%. We further investigated the sensitivity to lower and higher resistance test costs (single-gene sequencing) by varying the cost of the resistance test between £50 and £250. We investigated the sensitivity to the SVR12 after first-line treatment in NS5A-resistant patients treated for 12 weeks to determine the SVR12 threshold that would be required to ensure *Test12/8wks* is cost-effective. As there is no established evidence on the improvement in quality of life of patients with mild/moderate liver fibrosis after SVR, we assessed the sensitivity to this assumption by removing the utility increase (of 0.05) and assuming the same utilities for HCV-cleared and -infected patients.

Finally, we conducted a 2-way probabilistic sensitivity analysis on first-line treatment and retreatment drug prices to further explore the issue of drug costs and determine the most cost-effective strategy for a complete range of price combinations. Here, we considered differential percentage reductions in the cost of first- and second-line treatment.

## Results

### Base-Case Findings

Compared with *NoTest12wks*, *Test12/8wks* generated lower expected lifetime costs due to reduced treatment costs in nonresistant patients and higher expected QALY gains due to more favorable first- and second-line cure rates in nonresistant patients treated for 8 weeks (Table 2). These patients were also exposed to the disutility of treatment for a shorter period of time versus *NoTest12wks*. Nevertheless, *NoTest8wks* had the lowest expected lifetime costs due to lower treatment costs overall and higher QALY gains versus *NoTest12wks* due to the shortened exposure to the side effects of treatment. At £20 000 willingness-to-pay, *NoTest8wks* had an INMB of £12 289 (95% CrI £10 439-£14 100) and 74% probability of being the most cost-effective option; *Test12/8wks* had a lower INMB at £11 700 (95% CrI £10 439-£13 334) and only 26% probability of being the most cost-effective strategy. At £30 000 willingness-to-pay, the probability that *Test12/8wks* was the most cost-effective strategy was marginally higher, at 31%. At both willingness-to-pay thresholds, *NoTest8wks* was the optimal strategy with the highest expected INMB, and this finding was associated with a high level of certainty, as shown in the CEAC and CEAF (Appendix 3).

**Table 2:** Cost-effectiveness findings.

	Costs	QALYs	£20 000 WTP			£30 000 WTP		
			e(NMB)∗	e(INMB)∗	*P*(CE)	e(NMB)∗	e(INMB)∗	*P*(CE)
	(95% CrI)	(95% CrI)		(95% CrI)			(95% CrI)	
Base-case analysis								
NoTest12wks	£43 976 (£42 150-£46 470)	15.515 (15.011-16.167)	£266 319 (£254 664-£280 379)	—	.00	£421 467 (£404 872-£442 028)	—	.00
NoTest8wks	£31 698 (£29 744-£34 227)	15.515 (15.01-16.167)	£278 608 (£266 905-£292 679)	£12 289 (£10 439-£14 100)	.74	£433 761 (£417 198-£454 346)	£12 294 (£10 411-£14 142)	.69
Test12/8wks	£32 552 (£30 731-£34 982)	15.529 (15.021-16.182)	£278 019 (£266 301-£292 130)	£11 700 (£10 074-£13 334)	.26	£433 305 (£416 594-£453 941)	£11 838 (£10 183-£13 505)	.31
Sensitivity analysis (80% reduction in drug prices)								
NoTest12wks	£12 053 (£10 591-£14 268)	15.510 (15.008-16.165)	£298 150 (£286 460-£312 194)	—	.00	£453 252 (£436 581-£473 817)	—	.00
NoTest8wks	£9399 (£7946-£11 612)	15.511 (15.009-16.166)	£300 815 (£289 139-£314 856)	£2665 (£2194-£3116)	.55	£455 923 (£439 265-£476 535)	£2671 (£2163-£3157)	.33
Test12/8wks	£9683 (£8242-£11 851)	15.524 (15.018-16.181)	£300 795 (£289 020-£314 850)	£2645 (£2224-£3064)	.45	£456 034 (£439 299-£476 640)	£2782 (£2307-£3240)	.67

CrI indicates credible interval; e(INMB), expected incremental net monetary benefit; *P*(CE), probability most cost-effective; QALYs, quality-adjusted life years; WTP, willingness-to-pay. *NoTest12wks*, standard 12-week treatment duration (with no testing); *NoTest8wks*, shortened 8-week treatment duration (with no testing); *Test12/8wks*, baseline testing with 12-week treatment duration if NS5A resistant, 8 weeks otherwise.

∗ Versus NoTest12wks.

The number of liver-related events was relatively comparable across each strategy (Appendix 2). *Test12/8wks* had fewer cases of hepatocellular carcinoma, decompensated cirrhosis, and liver transplant than *NoTest12wks*, whereas *NoTest8wks* had slightly more; however, there was limited evidence to suggest the number of clinical events differed meaningfully across the strategies.

### Sensitivity/Scenario Analyses Findings

When drug prices were reduced by 80%, *Test12/8wks* became more cost-effective at £30 000 willingness-to-pay, as the small improvement in QALY gains was cost-effective at the lower drug tariff and higher cost-effectiveness threshold (Table 2). *Test12/8wks* had an expected INMB of £2782 (95% CrI £2307-£3240) and 67% probability of being the most cost-effective strategy; *NoTest8wks* had an INMB of £2671 (95% CrI £2163-£3157) and 33% probability of being the most cost-effective strategy. At the lower cost-effectiveness threshold of £20 000, *NoTest8wks* had the highest probability of being most cost-effective (55%), although there was more uncertainty around the optimal strategy (see CEAC and CEAF in Appendix 3).

**Figure 1:**
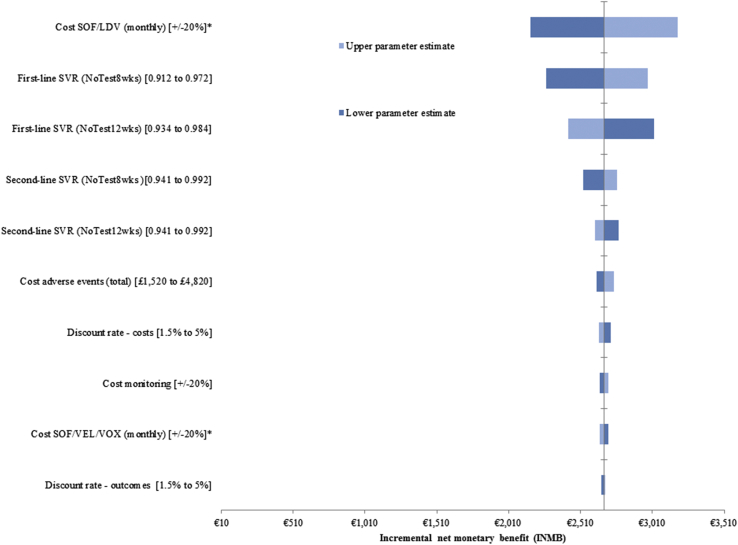
One-way sensitivity analysis of *NoTest8wks* versus *NoTest12wks.*

^∗^Assumes 80% reduction in drug costs. *NoTest12wks* indicates standard 12-week treatment duration (with no testing); *NoTest8wks*, shortened 8-week treatment duration (with no testing).

**Figure 2:**
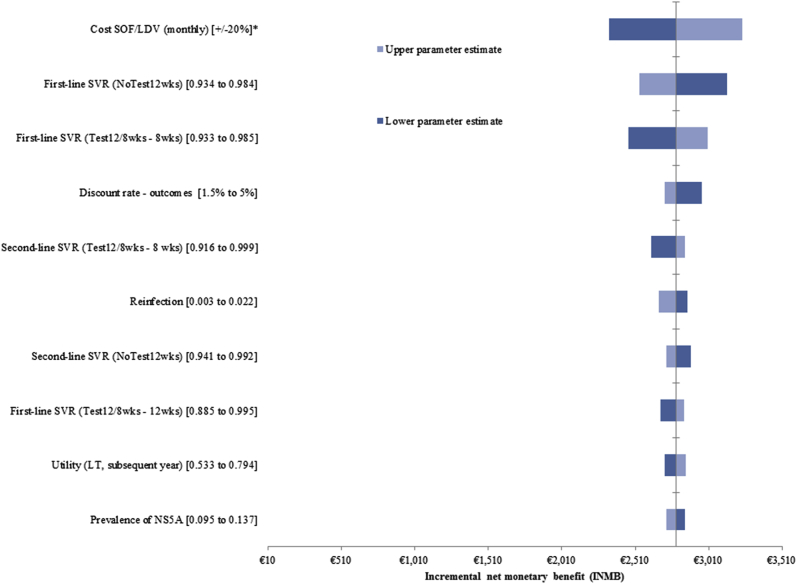
One-way sensitivity analysis of *Test12/8*wks versus *NoTest12wks.*

^∗^Assumes 80% reduction in drug costs. *NoTest12wks* indicates standard 12-week treatment duration (with no testing); *Test12/8wks*, baseline testing with 12-week treatment duration if NS5A resistant, 8 weeks otherwise.

Figures 1 and 2 present the top 10 varied parameters of the 1-way sensitivity analysis of *NoTest8wks* and *NoTest12/8wks* versus *NoTest12wks*, respectively; the full complement of results is presented in Appendix 4. Both strategies remained cost-effective when parameters were held at their upper/lower bounds. The key drivers of cost-effectiveness were the cost and efficacy of first-line treatment. Lower first-line treatment costs reduced the expected INMB of both strategies relative to *NoTest12wks* because of the reduced cost-savings overall. The INMB of *NoTest8wks* and *Test12/8wks* increased when SVR, after first-line treatment, was held at its upper value (97.2% and 98.5% in nonresistant patients treated for 8 weeks, respectively). The cost-effectiveness of both strategies also increased when SVR in the *NoTest12wks* strategy was held at its lower value (93.3%). Few other inputs had an effect on the INMB of *NoTest8wks* and *NoTest12/8wks*.

**Figure 3:**
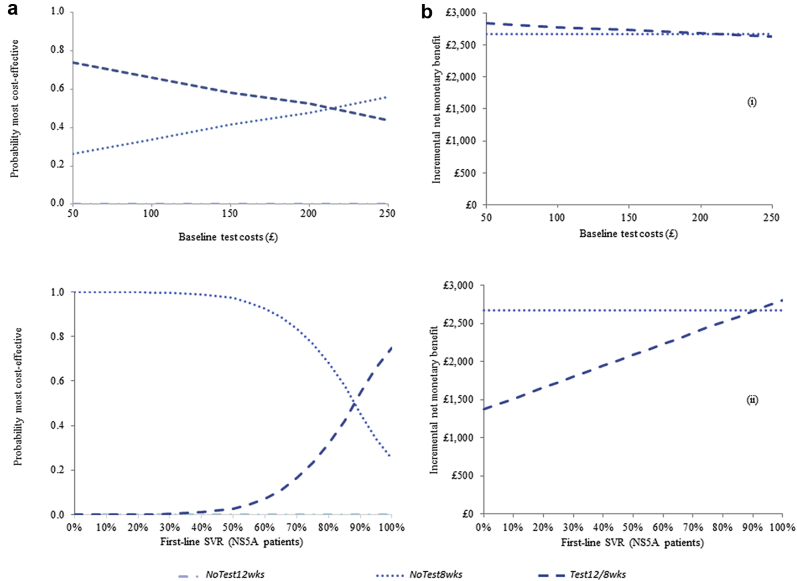
Results on the (a) probability of cost-effectiveness and (b) expected incremental net monetary benefit versus 12 weeks (no testing) of various scenario analyses, at £30 000 willingness-to-pay: (i) different resistance test costs (assuming 80% reduction in drug costs) and (ii) different first-line cure rates in patients with NS5A resistance (assuming 80% reduction in drug costs).

Findings from the scenario analyses of different resistance test costs and different first-line cure rates in patients with NS5A resistance are presented in Figure 3. The probability of cost-effectiveness and expected INMB are presented for each scenario at £30 000 willingness-to-pay. In each scenario, the probability of cost-effectiveness and expected INMB are presented on the vertical axis, with changes in the key parameter outlined on the horizontal axis. Results were somewhat sensitive to increases in the cost of the resistance test (ie, single-gene sequencing test). At resistance test costs greater than £220 (and assuming an 80% reduction in drug prices), *Test12/8wks* was no longer the most cost-effective strategy (base-case cost was £100); at these costs, *NoTest8wks* had the highest probability (56%) of being most cost-effective. Nevertheless, little difference in the expected INMB versus *NoTest12wks* was observed, with both strategies proving cost-effective. Results were sensitive to variations in the first-line cure rate in patients with NS5A resistance. At cure rates less than 87.5% over 12 weeks, *Test12/8wks* was no longer the most cost-effective strategy (base-case SVR12 was 95.7%), losing out to *NoTest8wks*, which had lower overall lifetime costs. Nevertheless, *Test12/8wks* remained more cost-effective than *NoTest12wks*, returning a positive expected INMB at all SVR rates below the base case.

*NoTest12wks* indicates standard 12-week treatment duration (with no testing); *NoTest8wks*, shortened 8-week treatment duration (with no testing); *Test12/8wks*, baseline testing with 12-week treatment duration if NS5A resistant, 8 weeks otherwise.

The results remained generally unchanged when we assumed the same utilities for HCV-cleared as HCV-infected patients, as detailed in Appendix 5.

**Figure 4:**
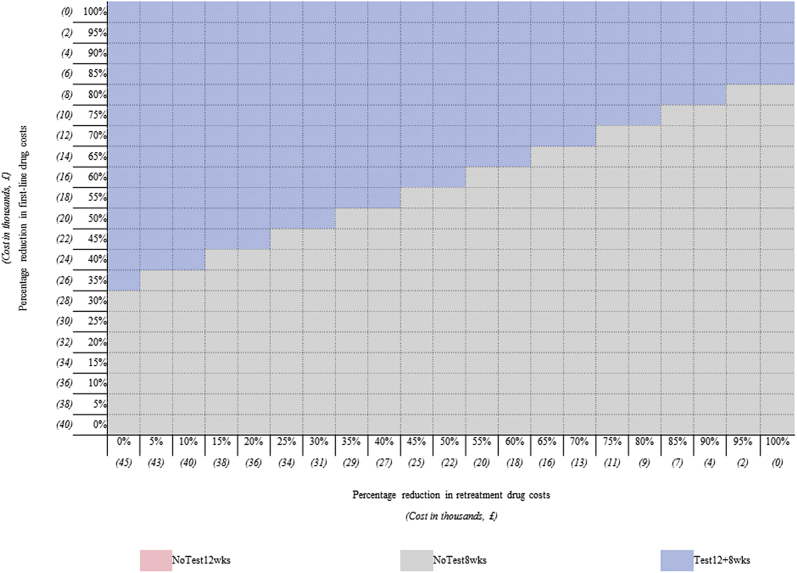
Results of the probability of cost-effectiveness of differential percentage reductions in the cost of first-line treatment and retreatment, at £30 000 willingness-to-pay.

Finally, Figure 4 presents the results from the 2-way probabilistic sensitivity analysis that compared differential percentage reductions in the cost of first-line treatment and retreatment. The percentage reduction in the cost of first-line treatment is presented on the *y*-axis, with the percentage reduction in the cost of retreatment depicted on the *x*-axis. The grid reports the probability that any strategy is the most cost-effective strategy for a given price combination (ie, percentage reduction). When first-line treatment drug prices were low (<£20k) and retreatment drug costs were high (>£22k), the findings favored *Test12/8wks*. Conversely, when retreatment drug costs were low (<£22k) and first-line treatment drug prices were high (>£20k), the results favored *NoTest8wks.* When both first-line treatment and retreatment drug costs were less than <£20k, the results largely favored *Test12/8wks*; however, some uncertainty was observed. Increased percentage reductions in the cost of retreatment sometimes favored *NoTest8wks*, as the strategy had a greater number of patients requiring retreatment. Hence, the strategy benefitted from reductions in the cost of the salvage regimen. For no given price combination was *NoTest12wks* cost-effective.

*NoTest12wks* indicates standard 12-week treatment duration (with no testing); *NoTest8wks*, shortened 8-week treatment duration (with no testing); *Test12/8wks*, baseline testing with 12-week treatment duration if NS5A resistant, 8 weeks otherwise. Under no scenario did *NoTest12wks* have the highest probability of being most cost-effective.

## Discussion

### Strengths and Limitations

This is the first study to consider the cost-effectiveness of testing for resistance to NS5A inhibitor in the United Kingdom. Furthermore, it is the first study to consider it in the context of stratifying patients to a shortened 8-week treatment duration in the absence of NS5A polymorphisms. Previous analyses that considered baseline testing used longer treatment durations, with patients stratified to a 12-week treatment duration in the absence of NS5A polymorphisms or 16-week treatment duration in the presence of NS5A polymorphisms, which is more costly and often of limited clinical benefit.^8^ Elbasha and colleagues^7^ also considered longer durations; however, the authors investigated the cost-effectiveness of baseline testing in patients who generally require extended treatment durations because of severe liver fibrosis or compensated cirrhosis. In GT1 noncirrhotic TN patients, an 8-week treatment duration is effective^5^^,^^9^ and cost-effective across a range of DAAs currently approved for use in the United Kingdom.^10^ The shortened treatment duration is becoming more commonplace, with newer regimens now being administered over 8 weeks.^11^ Nevertheless, our findings suggest that where significant price reductions are available, the nonstratified treatment approach (*NoTest8wks*) is less favorable than the stratified/personalized treatment approach (*Test12/8wks*) considered here.

We used LDV/SOF as our first-line treatment regimen because of the availability of data on the effectiveness of the regimen over a shortened 8-week treatment duration and by NS5A resistance. Our findings may be generalizable to newer regimens, such as ELB/GZR and SOF/VEL, which carry similar costs and health utilities, if the cure rates produced by these regimens are comparable with LDV/SOF over the same treatment durations and by NS5A resistance. At present, these regimens are licensed for 12 weeks in the United Kingdom,^6^^,^^29^ so there is limited evidence on the effectiveness of these regimens over the shortened treatment duration and by NS5A resistance. It is likely, however, that comparable cure rates may be achieved with the newer regimens, suggesting our findings are not limited to use of LDV/SOF but can be applied to other commonly used DAAs currently licensed for 12 weeks. Whether resistance testing is preferable to an 8- or 12-week treatment duration largely depends on the price combination of first- and second-line DAA therapy, which we report in full in this analysis.

There are limitations associated with this work. Although we used rich data from Sarrazin and colleagues^5^ on the prevalence of NS5A resistance among genotype 1 noncirrhotic TN patients, we acknowledge these data, which were derived from clinical trials in Europe and the United States, may not reflect the prevalence of NS5A resistance in the UK population. We combined information on subtypes GT1a and GT1b in our analysis and assumed the same outcomes (SVR) in patients with NS5A polymorphisms across the 2 subtypes. It is possible that outcomes differ between GT1a and GT1b. Sarrazin et al^5^ observed a slightly higher SVR in GT1a than GT1b TN patients; however, the authors could discern no significant difference between the 2 subtypes. We grouped NS5A RASs (ie, Q30H, Q30G, Q30R, etc) in our analysis, as in Sarrazin et al,^5^ but further stratification by RASs could be undertaken. Nevertheless, such an analysis requires a detailed breakdown of SVR by each polymorphism, which has yet to be undertaken or made available, to the best of our knowledge. We explored the cost-effectiveness of testing for NS5A resistance, but other resistance variants exist (eg, NS5B and NS3) and could be explored with further research. Nevertheless, Sarrazin et al^5^ found no association between these variants and treatment outcomes. The Markov model assumed yearly cycles to reflect the known natural disease history of HCV. A limitation of using yearly cycles is that the model could not capture the probability of 2 events occurring in the same year, such as progression to compensated cirrhosis and hepatocellular carcinoma. The probability of these events occurring, however, particularly hepatocellular carcinoma, is low in this population of noncirrhotic patients, and few patients progress to compensated cirrhosis overall because of the highly efficacious nature of first- and second-line treatment. Shorter cycles could be adopted but are unlikely to detect meaningful differences between the strategies, which had similarly low numbers of clinical events. The model assumed that patients who failed first-line therapy were quickly retreated and that there was no loss to follow-up. Nevertheless, in practice, second-line therapy may be delayed, leading to a potential loss to follow-up, particularly in problematic populations, such as chaotic drug users or prison inmates serving short sentences. Future research should explore the potential cost-effectiveness of baseline testing in these specific populations.

### Implications for Practice

The clinical and practical benefits of stratifying patients to different treatment durations based on the presence of NS5A polymorphisms are clear. Patients’ chances of viral eradication are increased, and for any patient failing first-line therapy, their future likelihood of viral eradication with retreatment is high, with success rates in excess of 96% observed, even in patients with NS5A resistance.^14^ Treating most patients over a shortened 8-week treatment duration provides an opportunity not only to address the burden of high treatment costs that arise with longer treatment durations but also better deliver care to more patients quicker. This may be particularly relevant in health systems where the cost of HCV drugs remains a barrier to wider access. Aside from cost, the main negative issue related to resistance testing is the time taken and degree of specialization needed to receive and interpret results. In some settings, this may present an obstacle to increasing access to treatment, particularly in high-burden/low-income settings where infrastructure is limited. In the United Kingdom, testing for resistance to NS5A inhibitors at baseline is routinely recommended for patients receiving ELB/GZR, so the infrastructure exists to expand this to all patients.

Testing for resistance to NS5A inhibitor-containing regimens in GT1 noncirrhotic TN patients in the United Kingdom is cost-effective as drug prices fall to lower levels. The personalized approach to treatment offers significant clinical and practical benefits: the patient’s chances of viral eradication are maximized, fewer patients progress to more advanced stages of liver disease, and more patients can be effectively treated quicker.

## Conclusions

We investigated the cost-effectiveness of testing for resistance to NS5A inhibitor-containing regimens at baseline, with treatment duration optimized according to the presence of NS5A polymorphisms, in GT1 noncirrhotic TN patients in the United Kingdom. The cost of treatment proved the key driver of cost-effectiveness in this study. Using cost-effectiveness thresholds of £20 000 to £30 000, we found that baseline testing (*Test12/8wks*) has a low probability (26%-31%) of being cost-effective when the cost of first-line treatment and retreatment are high (∼£40 000 and ∼£44 000 per patient over 12 weeks, respectively). At these prices, a shortened 8-week treatment duration (*NoTest8wks*) has the highest probability (69%-74%) of being most cost-effective. Nevertheless, when drug prices are reduced by 80%, the results are reversed at the higher £30 000 cost-effectiveness threshold. In fact, we found baseline testing has the highest probability of being most cost-effective when first-line treatment drug prices are low (<£20 000) and retreatment drug prices are high (>£22 000). When both first-line treatment and retreatment drug prices are low (<£20 000), baseline testing largely remains the most cost-effective strategy; however, shortened 8-week treatment is sometimes favored, as the strategy benefits more from increased price reductions in the cost of retreatment because of greater numbers requiring the salvage regimen. Although the cost of first-line treatment and retreatment remains unknown, we present the complete range of price combinations that could exist and report the combinations at which the proposed strategies have the highest probability of being most cost-effective, provided society is willing to pay £30 000 per QALY gained.

The results are somewhat sensitive to increases in the cost of the resistance test. Baseline testing is most cost-effective up to a threshold of £220 per resistance test (assuming an 80% reduction in drug prices). Single-gene sequencing is expected to cost in the region of £50 to £150 in the United Kingdom, suggesting the strategy is cost-effective at the lower drug tariffs. The strategy is also sensitive to variations in SVR12 in patients with NS5A resistance treated over 12 weeks (assuming the same 80% reduction in drug prices). At cure rates less than 87.5%, the strategy is no longer the most cost-effective option. Nevertheless, DAAs rarely produce cure rates less than 90% over a 12-week treatment duration, suggesting that this threshold is unlikely to be breached. Overall, we found that the standard 12-week treatment (*NoTest12wks*) is not cost-effective versus either baseline testing or a shortened 8-week treatment.

## Supplementary Material

Appendix 1 Model structure Appendix 2 – Number of clinical events (avoided) Appendix 3 – Cost-effectiveness acceptability curves and cost-effectiveness acceptability frontier; base case and sensitivity analysis Appendix 4 – Complete results of the 1-way sensitivity analyses for *NoTest8wks* and *Test12/8wks* versus *NoTest12wks.* Appendix 5 – Sensitivity analysis with increment in utility after sustained virological response excluded
