# Supplementary material for: Cost-Effectiveness Analysis of Baseline Testing for Resistance-Associated Polymorphisms to Optimize Treatment Outcome in Genotype 1 Noncirrhotic Treatment-Naïve Patients With Chronic Hepatitis C Virus
Source: Value Health. 2020 Feb;23(2):180–90. doi: 10.1016/j.jval.2019.08.012 (PMC7057278; doi:10.1016/j.jval.2019.08.012)
Supplement: Appendix 1 — Model structure Appendix 2 – Number of clinical events (avoided) Appendix 3 – Cost-effectiveness acceptability curves and cost-effectiveness acceptability frontier; base case and sensitivity analysis Appendix 4 – Complete results of the 1-way sensitivity analyses for NoTest8wks and Test12/8wks versus NoTest12wks. Appendix 5 – Sensitivity analysis with increment in utility after sustained virological response excluded [file mmc1.docx]

# Cost-effectiveness analysis of baseline testing for resistance-associated polymorphisms to optimise treatment outcome in genotype 1 non-cirrhotic treatment-naïve patients with chronic hepatitis c virus

Supplementary material

# Appendix 1 – Model structure


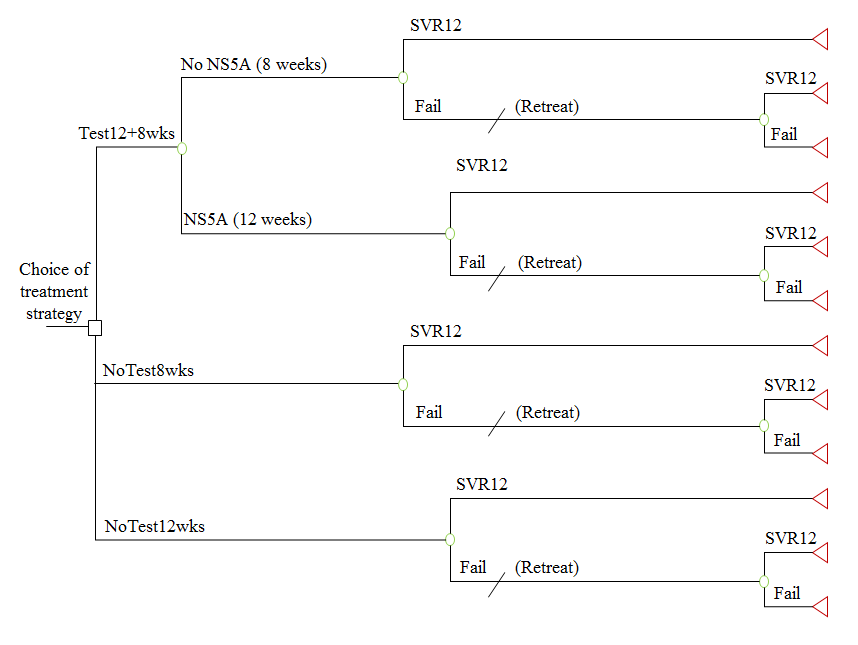
 **Figure 1** Decision tree simulating treatment outcomes

NS5A, non-structural protein 5A; SVR12, sustained virologic response at 12 weeks post end of treatment

*NoTest12wks*: ‘standard 12 weeks treatment duration (with no testing)’;

*NoTest8wks*: ‘shortened eight weeks treatment duration (with no testing)’;

*Test12/8wks*: ‘baseline testing’ with 12 weeks treatment duration if NS5A resistant, eight weeks otherwise


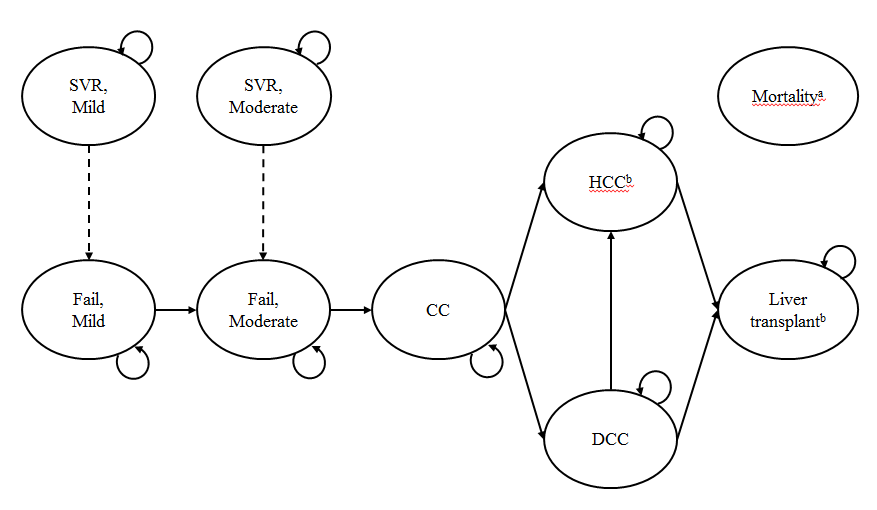


**Figure 2** Markov model simulating natural disease history

Patients entered the model depending on their response to treatment (SVR or fail) and liver fibrosis (mild or moderate)

-- Dotted line reflects reinfection

^a^ Captures all-cause mortality and liver-related mortality

^b^ Models first and subsequent years to reflect different liver-related mortality rates

CC, compensated cirrhosis; DCC, decompensated cirrhosis; HCC, hepatocellular carcinoma; SVR, sustained virologic response

# Appendix 2 – Number of clinical events (avoided)

| **Table 1** Number of events and events avoided | | |  |  |  |  |
| --- | --- | --- | --- | --- | --- | --- |
|  | HCC  (95% CI) | DCC  (95% CI) | LT  (95% CI) | HCC avoided (95% CI) | DCC avoided (95% CI) | LT avoided (95% CI) |
| NoTest12wks | 16.204  (2.392 to 47.875) | 39.241  (10.634 to 86.002) | 3.854  (1.02 to 8.448) | - | - | - |
| NoTest8wks | 16.244  (2.4 to 47.879) | 39.338  (10.669 to 86.066) | 3.864  (1.032 to 8.484) | -0.040  (-0.291 to 0.155) | -0.097  (-0.628 to 0.343) | -0.011  (-0.069 to 0.038) |
| Test12/8wks | 16.194  (2.388 to 47.776) | 39.216  (10.582 to 85.893) | 3.851  (1.024 to 8.464) | 0.010  (-0.218 to 0.214) | 0.025  (-0.475 to 0.465) | 0.003  (-0.052 to 0.051) |

DCC, decompensated cirrhosis; HCC, hepatocellular carcinoma; SVR, sustained virologic response

*NoTest12wks*: ‘standard 12 weeks treatment duration (with no testing)’;

*NoTest8wks*: ‘shortened eight weeks treatment duration (with no testing)’;

*Test12/8wks*: ‘baseline testing’ with 12 weeks treatment duration if NS5A resistant, eight weeks otherwise

# Appendix 3 – Cost-effectiveness acceptability curves and cost-effectiveness acceptability frontier – base case and sensitivity analysis

**Figure 1** Probability of cost-effectiveness at different willingness-to-pay thresholds (base case analysis)

**Figure 2** Cost-effectiveness acceptability frontier at different willingness-to-pay thresholds (base case analysis)

*NoTest12wks*: ‘standard 12 weeks treatment duration (with no testing)’;

*NoTest8wks*: ‘shortened eight weeks treatment duration (with no testing)’;

*Test12/8wks*: ‘baseline testing’ with 12 weeks treatment duration if NS5A resistant, eight weeks otherwise

**Figure 3** Probability of cost-effectiveness at different willingness-to-pay thresholds (sensitivity analysis with 80% reduction in drug prices)

**Figure 4** Cost-effectiveness acceptability frontier at different willingness-to-pay thresholds (sensitivity analysis with 80% reduction in drug prices)

*NoTest12wks*: ‘standard 12 weeks treatment duration (with no testing)’;

*NoTest8wks*: ‘shortened eight weeks treatment duration (with no testing)’;

*Test12/8wks*: ‘baseline testing’ with 12 weeks treatment duration if NS5A resistant, eight weeks otherwise

# Appendix 4 – Complete results of the one-way sensitivity analyses for NoTest8wks and Test12/8wks versus NoTest12wks

* Assumes 80% reduction in drug costs

**Figure 1** One-way sensitivity analysis of NoTest8wks versus NoTest12wks

* Assumes 80% reduction in drug costs

**Figure 2** One-way sensitivity analysis of Test12/8wks versus NoTest12wks

*NoTest12wks*: ‘standard 12 weeks treatment duration (with no testing)’;

*NoTest8wks*: ‘shortened eight weeks treatment duration (with no testing)’;

*Test12/8wks*: ‘baseline testing’ with 12 weeks treatment duration if NS5A resistant, eight weeks otherwise

# Appendix 5 – Sensitivity analysis with increment in utility following SVR excluded

| **Table 1** Sensitivity analysis excluding 0.05 increment in utility following SVR | | | | | | |
| --- | --- | --- | --- | --- | --- | --- |
|  |  |  | £20,000 WTP | | £30,000 WTP | |
|  | Costs  (95% CrI) | QALYs  (95% CrI) | INMB  (95% CrI)^a^ | p(CE)  (95% CrI)^a^ | INMB  (95% CrI)^a^ | p(CE)  (95% CrI)^a^ |
| NoTest12wks | £12,034  (£10,544 to £14,229) | 14.618  (14.218 to 15.181) | - | 0.00 | - | 0.00 |
| NoTest 8weeks | £9,383  (£7,874 to £11,614) | 14.619  (14.219 to 15.182) | £2,672  (£2,231 to £3104) | 0.57 | £2,682  (£2,214 to £3,131) | 0.35 |
| Test12/8wks | £9,672  (£8,192 to £11,924) | 14.632  (14.227 to 15.198) | £2,635  (£2,230 to £3038) | 0.43 | £2,772  (£2,323 to £3,211) | 0.65 |

^a^ Versus ‘NoTest12wks’

CrI, credible interval; e(INMB), expected incremental net monetary benefit; p(CE), probability most cost-effective; QALYs, quality-adjusted life years; WTP, willingness-to-pay

*NoTest12wks*: ‘standard 12 weeks treatment duration (with no testing)’;

*NoTest8wks*: ‘shortened eight weeks treatment duration (with no testing)’;

*Test12/8wks*: ‘baseline testing’ with 12 weeks treatment duration if NS5A resistant, eight weeks otherwise

**Figure 1** Probability of cost-effectiveness at different willingness-to-pay thresholds of sensitivity analysis excluding 0.05 increment in utility following SVR

*NoTest12wks*: ‘standard 12 weeks treatment duration (with no testing)’;

*NoTest8wks*: ‘shortened eight weeks treatment duration (with no testing)’;

*Test12/8wks*: ‘baseline testing’ with 12 weeks treatment duration if NS5A resistant, eight weeks otherwise
